# Second Correction for Huang et al., “*In Vitro* and *In Vivo* Activities of Zinc Linolenate, a Selective Antibacterial Agent against *Helicobacter pylori*”

**DOI:** 10.1128/aac.01544-25

**Published:** 2026-01-27

**Authors:** Yanqiang Huang, Xudong Hang, Xueqing Jiang, Liping Zeng, Jia Jia, Yong Xie, Fei Li, Hongkai Bi

## AUTHOR CORRECTION

Volume 63, no. 6, e00004-19, 2019, https://doi.org/10.1128/aac.00004-19. Supplemental material: Fig. S10 should appear as shown in this correction. Upon review, we discovered an error in panel A of Fig. S10 in which the H&E-stained image of the stomach was incorrectly placed during the file organization process. The correction does not change any of the conclusions drawn in the study and does not refer to the previous correction. We sincerely apologize for any confusion this may have caused.

## SUPPLEMENTAL MATERIAL

Supplemental material aac.01544-25-s0001.pdf Supplement file

ASM does not own the copyrights to Supplemental Material that may be linked to, or accessed through, an article. The authors have granted ASM a non-exclusive, world-wide license to publish the Supplemental Material files. Please contact the corresponding author directly for reuse.

