# Supplementary material for: Second Correction for Huang et al., “In Vitro and In Vivo Activities of Zinc Linolenate, a Selective Antibacterial Agent against Helicobacter pylori”
Source: Antimicrob Agents Chemother. 2026 Jan 27;70(3):e01544-25. doi: 10.1128/aac.01544-25 (PMC12959144; doi:10.1128/aac.01544-25)
Supplement: Supplemental material — Supplement file [file aac.01544-25-s0001.pdf]

Supplement to:

In Vitro and *In Vivo* Activities of Zinc Linolenate, a Selective Antibacterial Agent against *Helicobacter pylori*

Yanqiang Huang[a], Xudong Hang[a], Xueqing Jiang[a], Liping Zeng[a], Jia Jia[a], Yong Xie[b], Fei Li[c], Hongkai Bi[a,#]

[a]Department of Pathogen Biology, Jiangsu Key Laboratory of Pathogen Biology, Nanjing Medical University, Nanjing, Jiangsu 211166, China

[b]Department of Gastroenterology, the First Affiliated Hospital of Nanchang University, Nanchang, Jiangxi 330006, China.

[c]Department of Medicinal Chemistry, School of Pharmacy, Nanjing Medical University, Nanjing, Jiangsu 211166, China.

Table S1. Broth microdilution MIC of metal complex of unsaturated fatty acid for *H. pylori* strains. (MIC: μg/ml)

**Strain**	**Drug sensitivity**	**BiLla**	**FeLla**	**ZnOa**	**ZnSa**	**ZnPa**	**ZnMa**
HP2665	S	128	>128	>128	>128	>128	>128
NSH57	S	64	>128	>128	>128	>128	>128
G27	S	64	>128	>128	>128	>128	>128
HP159	L, C, M (R)	128	>128	>128	>128	>128	>128
HP163	L, M (R)	64	>128	>128	>128	>128	>128
HP160	M (R)	64	128	>128	>128	>128	>128
HP161	C (R)	128	>128	>128	>128	>128	>128
HP162	L (R)	64	128	>128	>128	>128	>128

Abbreviations: L, levofloxacin; C, clarithromycin; M, metronidazole; S, Drug sensitive; R, Drug resistance; BiLla, bismush linolenate; FeLla, iron linolenate; ZnOa, zinc oleate; ZnSa, zinc stearate; ZnPa, zinc palmitate; ZnMa, zinc myristate.

Table S2. The toxicity of ZnLla after oral gavage.

Groups	ALT (U/L)	AST (U/L)	BUN (mmol/L)	CREA (μmol/L)
Infected (PBS)	22.00±4.36	110.00±14.73	13.90±3.72	16.67±1.15
Infected (OPZ+AC)	34.33±3.43	220.00±18.40	8.77±1.23	17.00±1.00
Infected (OPZ+ZnLla)	19.67±1.53	117.00±12.61	10.27±0.32	16.33±0.58
Infected (ZnLla)	20.00±4.12	182.33±14.74	14.97±3.28	18.33±1.06
Uninfected (10-fold dosage of ZnLla)	23.33±0.58	138.33±13.18	11.90±0.79	15.00±2.00
Uninfected (PBS)	29.00±6.93	231.00±17.25	13.13±1.42	18.33±1.69

Abbreviations: ALA, Alanine aminotransferase; AST, Aspartate aminotransferase; BUN, urea nitrogen; CREA, creatinine.

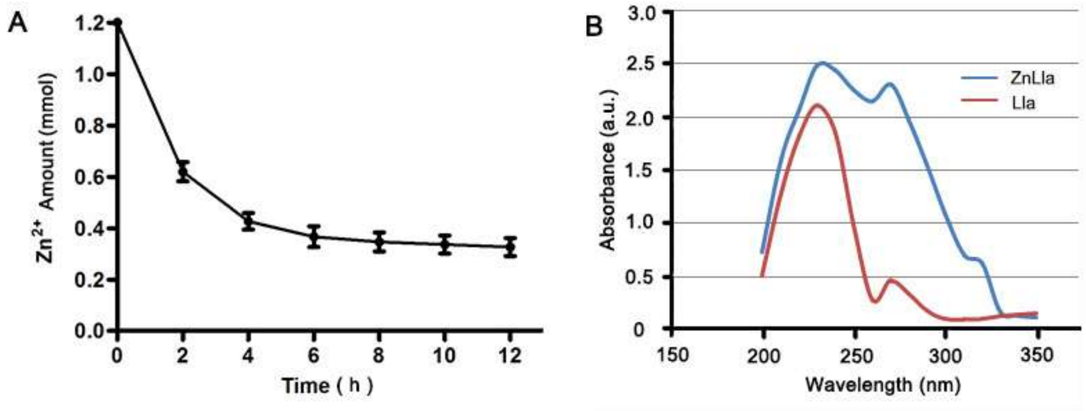

Figure S1. A. The amount change of Zn^{2+} during the reaction process of ZnLla. B. UV-Vis spectrum of ZnLla (blue line) and Lla (red line).

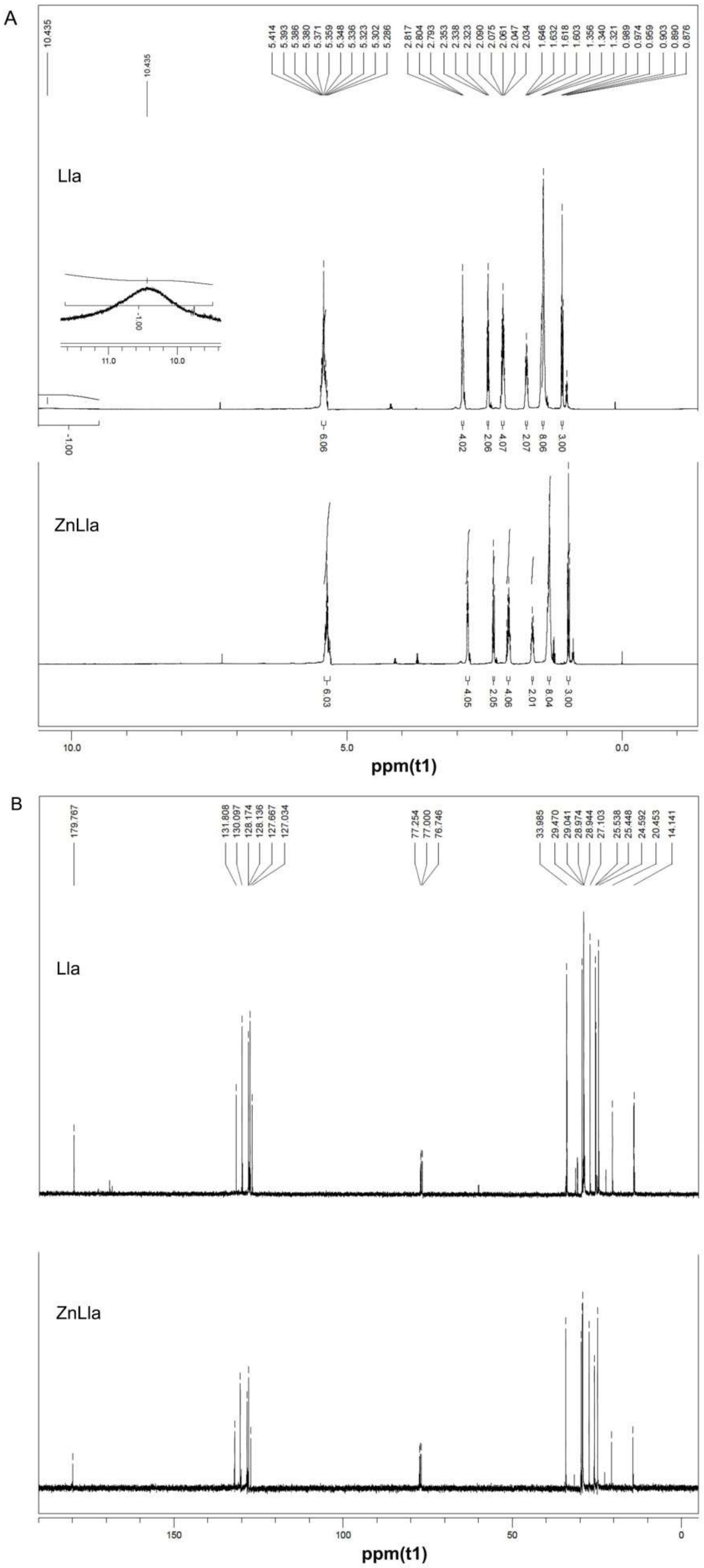
A
Lla
ZnLla
ppm(t1)
B
Lla
ZnLla
ppm(t1)

Figure S2. A. ^{1}H NMR spectrum of Lla and ZnLla in $CDCl_3$ (500 MHz, $CDCl_3$) , δ5.40 ~ 5.30 (m, 6H), 2.81 (t, J = 6.5 Hz, 4H), 2.34 (t, J = 7.5 Hz, 2H), 2.09 ~ 2.03 (m, 4H), 1.66 ~ 1.60 (m, 2H), 1.36 ~ 1.32 (m, 8H), 0.97 (t, J = 7.5 Hz, 3H)； B. ^{13}C NMR spectrum of Lla and ZnLla in $CDCl_3$ (125 MHz, $CDCl_3$), δ179.9, 132.0, 130.3, 128.4, 127.9, 127.2, 34.2, 29.7, 29.2, 29.1, 27.3, 25.7, 24.8, 20.6, 14.3.

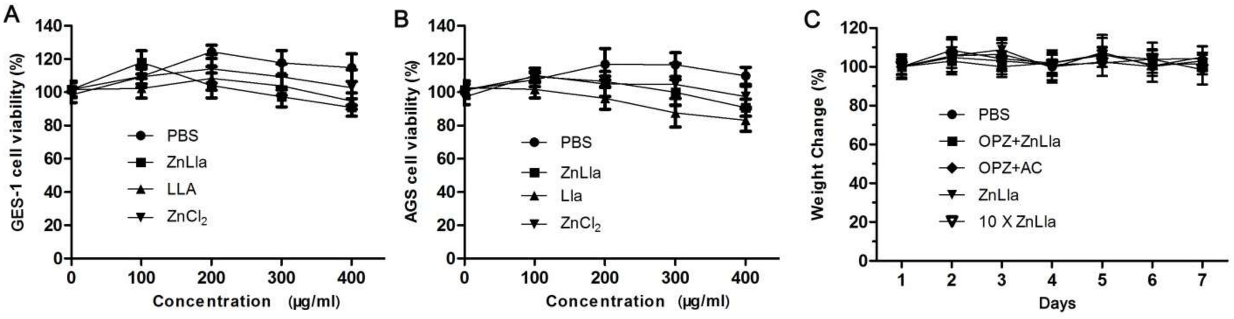

Figure S3. Cytotoxicity assays of ZnLla and effects on mouse weight. A. Cell viability test on GES-1 cells. B. Cell viability test on AGS cells. C. The effects on mice weight change.

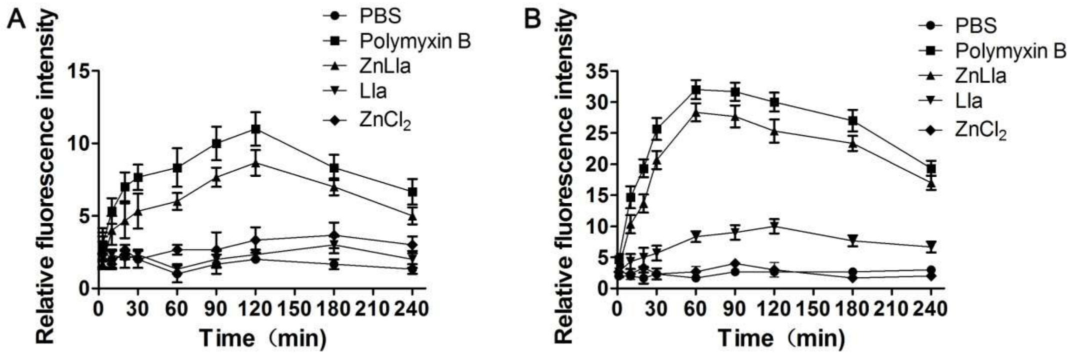

Figure S4. Uptake of N-phenylnapthylamine (NPN) by *H. pylori* G27 cells after treatment of PBS (negative control), 16 μg/ml (A) and 400 μg/ml (B) of polymyxin B (positive control), ZnLla, Lla or $ZnCl_2$.

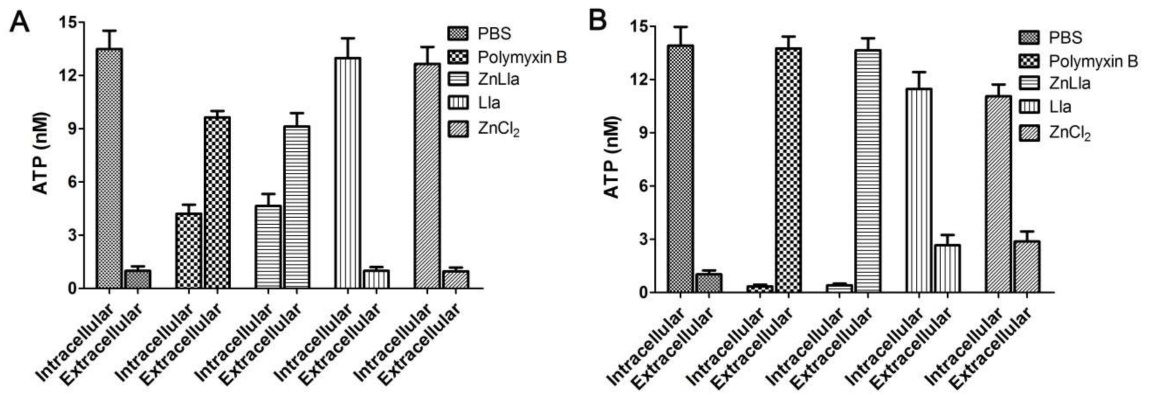

Figure S5. The release of ATP from *H. pylori* cells after treatment of PBS (negative control), 16 µg/ml (A) and 400 µg/ml (B) of polymyxin B (positive control), ZnLla, Lla or $ZnCl_2$.

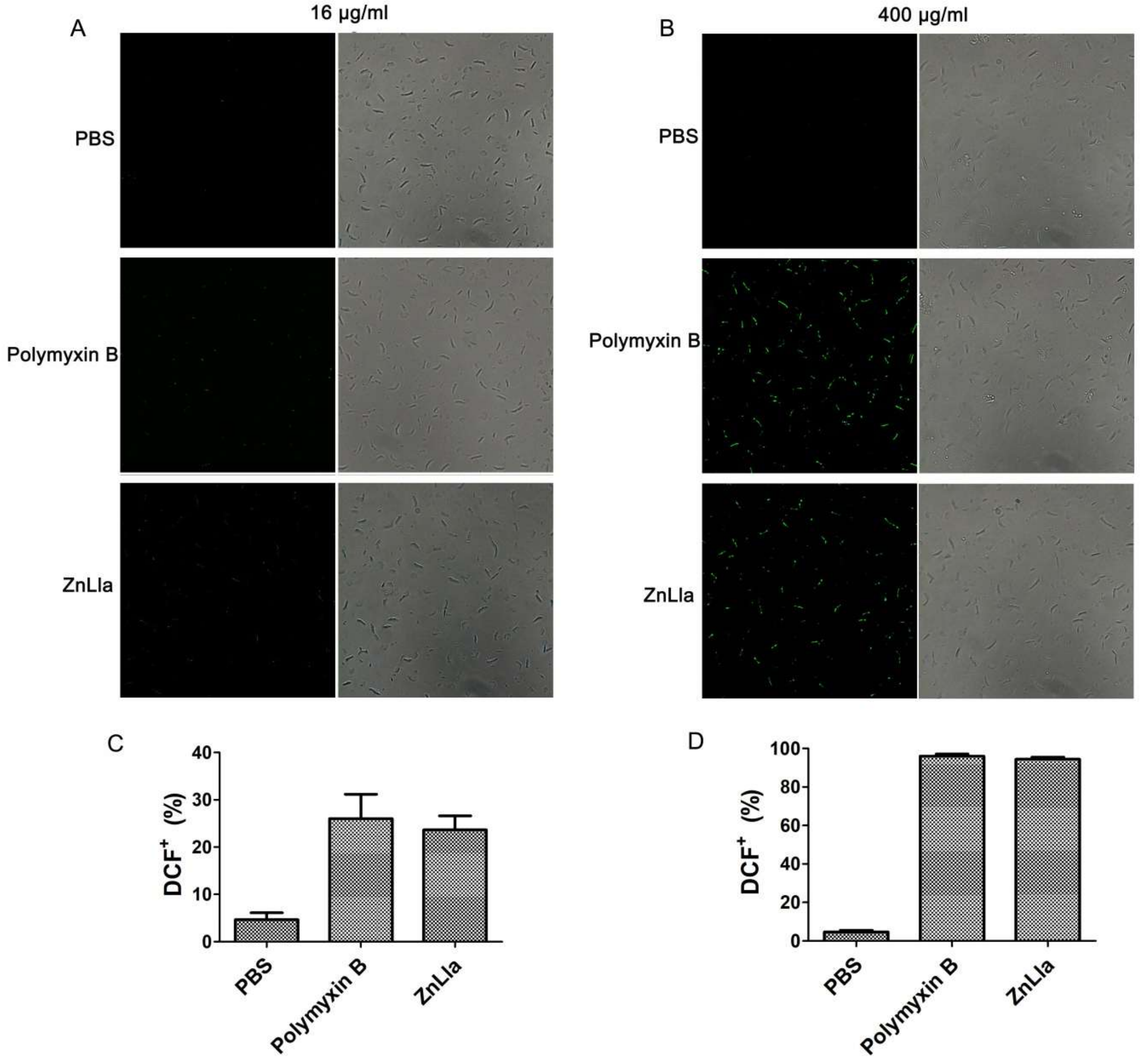

Figure S6. Fluorescence microscopic images (left) and bright field images (right) of DCF^+ labeled *H. pylori* stained with DCFDA after the incubation with PBS, 16 µg/ml (A) and 400 µg/ml (B) of polymyxin B or ZnLla. C and D. The ratios of DCF^+ cells incubated with each

treatment calculated by manually counting under a microscope. Data represent medians ± SD of three independent experiments.

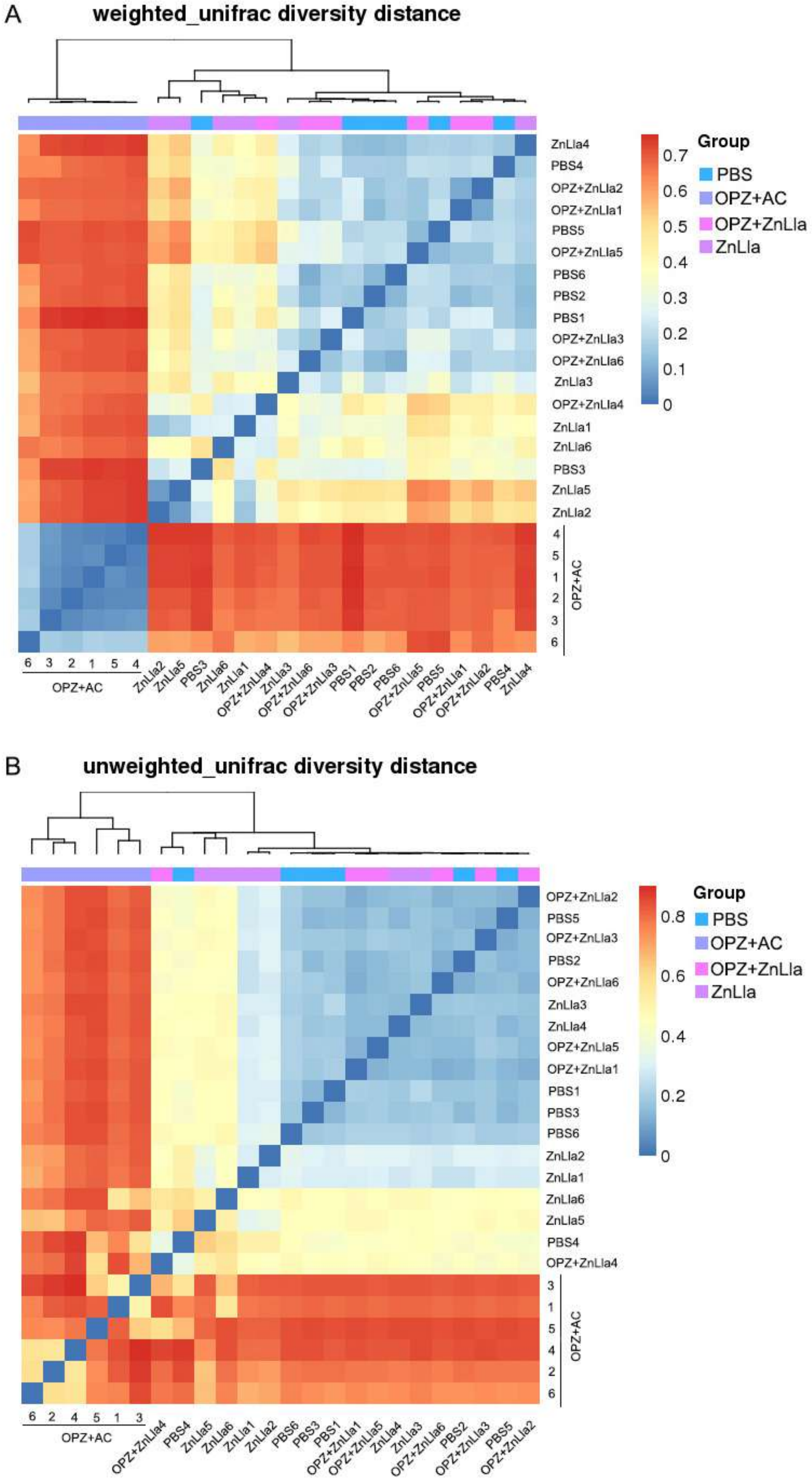

Figure S7. Beta diversity measurements of each sample receiving different treatments based on weighted (A) and unweighted (B) Unifrac analysis.

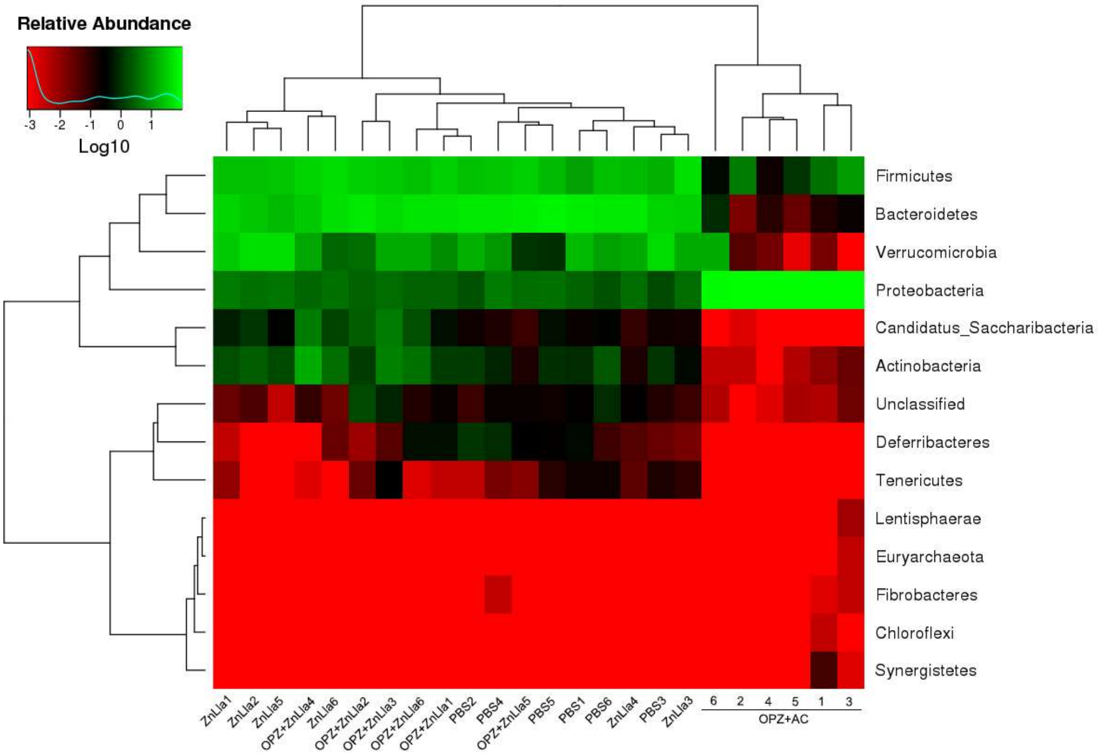

Figure S8. Log-scaled percentage heat map of Phylum-level in each sample receiving different treatments identified from the sequenced data.

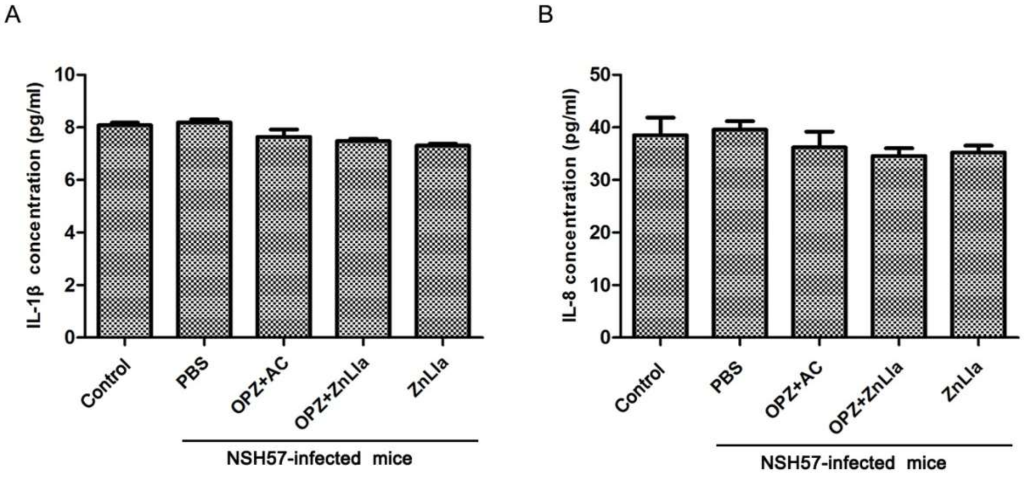

Figure S9. Proinflammatory cytokine production. Concentrations of serum IL-1β (A) and IL-8 (B) levels were determined by ELISA kits from NSH57-infected mice (Normal) and mice receiving different treatments.

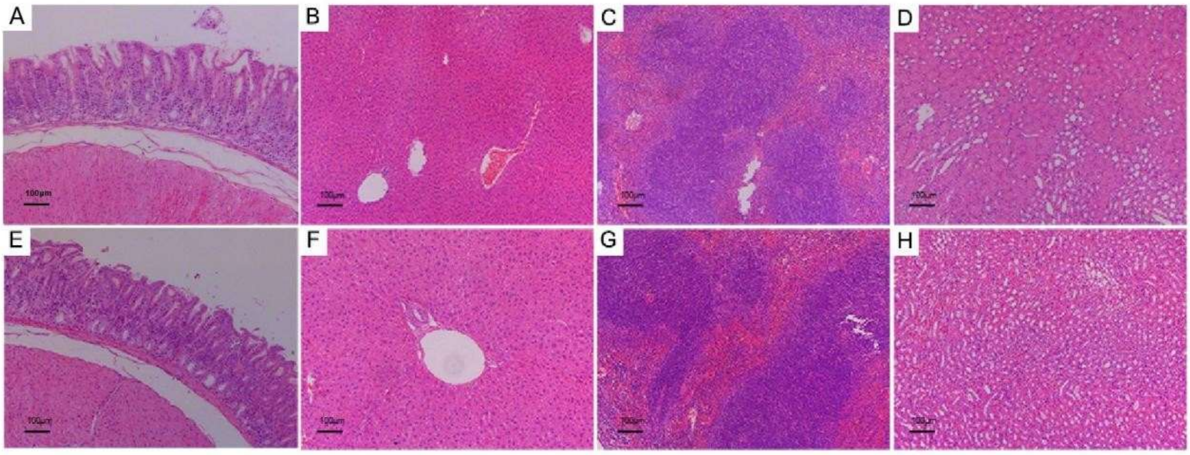

Figure S10. The H&E-stained stomach (A and E), liver (B and F), spleen (C and G) and kidney (D and H) from BHKS159-infected mice receiving 10-fold dosage of ZnLla (scale bar = 100 μm). A-D, ×100; E-H, ×200.
